# A human induced pluripotent stem cell model of Alzheimer’s Disease‐associated fractalkine receptor polymorphism to assess AD‐related microglial dysfunction

**DOI:** 10.1002/alz.089429

**Published:** 2025-01-03

**Authors:** Kaylee Tutrow, Jade Harkin, Melody Hernandez, Kang‐Chieh S Huang, Stephanie J Bissel, Shweta S Puntambekar, Bruce T. Lamb, Jason S. Meyer

**Affiliations:** ^1^ Indiana University School of Medicine, Indianapolis, IN USA; ^2^ Stark Neurosciences Research Institute, Indianapolis, IN USA; ^3^ Indiana University Purdue University Indianapolis, Indianapolis, IN USA; ^4^ Stark Neurosciences Research Institute, Indiana University School of Medicine, Indianapolis, IN USA

## Abstract

**Background:**

Dysfunctional microglial activity has recently been identified as a potential mechanism leading to accumulation of amyloid beta and pTau and subsequent neurodegeneration in Alzheimer’s Disease. The CX3CR1/fractalkine axis serves as a mechanism for bi‐directional communication between microglia and neurons, respectively, to promote a resting, anti‐inflammatory state in microglia. Previous studies have demonstrated that deficiency in CX3CR1 signaling leads microglia to a more pro‐inflammatory phenotype, phagocytic deficits, and increased susceptibility of neurons to cell death. Additionally, the CX3CR1‐V249I polymorphism was recently identified as a potential risk allele for Alzheimer’s Disease with worsened Braak staging in post‐mortem Alzheimer’s patients. However, the role of fractalkine dysfunction in human cells and the mechanisms by which microglia with the CX3CR1‐V249I SNP contribute to neurodegeneration remain unclear.

**Method:**

To address this shortcoming, we utilized human induced pluripotent stem cells and CRISPR/Cas9 gene editing technology to elucidate the effects of the CX3CR1‐V249I polymorphism on human microglia‐like cells (hMGLs) compared to an isogenic control cell line. Isogenic control cells alongside both heterozygous and homozygous CX3CR1 V249I cell lines were differentiated in parallel to yield enriched populations of hMGLs. Resulting hMGLs were then assessed for uptake of amyloid beta 1‐42 using flow cytometry, cell death in response to cytokine starvation, changes in proliferation, and finally alterations to migratory behavior using a microfluidic chamber.

**Result:**

We demonstrate the effective differentiation of hMGLS from both isogenic control and CX3CR1‐V249I backgrounds, which express characteristic microglial markers and are functionally phagocytic. Microglia bearing the homozygous CX3CR1‐V249I allele, but not heterozygous cells, demonstrated decreased uptake of amyloid beta in vitro compared to isogenic controls. Additionally, homozygous V249I microglia demonstrated increased stress‐induced cell death, as well as altered proliferation and decreased migratory capability.

**Conclusion:**

These findings suggest that the CX3CR1‐V249I polymorphism may cause a dysfunctional microglia phenotype that may contribute to neuronal dysfunction and death. Ongoing work will expand upon the transcriptome and secretome profile of CX3CR1‐V249I microglia and elucidate how this gene variant contributes to Alzheimer’s Disease‐related neurodegeneration.

